# Distribution characteristics and pollution assessment of heavy metals in typical black soil profiles of Haicheng city, Liaoning province, China

**DOI:** 10.1371/journal.pone.0314105

**Published:** 2025-01-24

**Authors:** Ziqi Li, Cang Gong, Xiaojun Ai, Xiaohuang Liu, Xiaofeng Zhao, Jiufen Liu

**Affiliations:** 1 China University of Geosciences, Beijing, China; 2 Natural Resources Comprehensive Survey Command Center of China Geological Survey, Beijing, China; 3 Research Center of Applied Geology of China Geological Survey, Chengdu, Sichuan, China; 4 Key Laboratory of Natural Resource Coupling Process and Effects, Beijing, China; 5 Center for Geophysical Survey, China Geological Survey, Langfang, Hebei, China; Makerere University College of Natural Sciences, UGANDA

## Abstract

In order to understand the spatial distribution, influencing factors, pollution level and sources of heavy metals in black soil profiles in Northeast China, black soil profile samples were collected from five sampling points in Haicheng City, Liaoning Province, with the deepest profile depth of 50m. The contents of heavy metals (As, Cd, Cr, Cu, Hg, Ni, Pb and Zn) in soil at different depths were analyzed, and the distribution characteristics and influencing factors of heavy metals in black soil profiles were analyzed. The pollution level of heavy metals in soil was evaluated based on the geo-accumulation index method and enrichment factor method, and the sources of heavy metals in soil were analyzed based on principal component analysis. The results show that the content ranges of As, Cd, Cr, Cu, Hg, Ni, Pb and Zn in the surface soil of the five profile sampling points are 7.74–16.5μg/g, 0.14–0.38μg/g, 75.4–104μg/g, 20.6–36.1μg/g, 0.031–0.20μg/g, 27.8–45.6μg/g, 28.5–45.6μg/g and 56.8–158μg/g, respectively. The Cd, Cr, Ni and Pb contents in the surface soil of the five profiles all exceeded the soil background values in Liaoning and China. Except for profile HCZK02, the contents of 8 heavy metals generally decrease with increasing depth. As the depth of profile HCZK02 increases, As, Hg and Pb show a decrease-increase-decrease change; Cd and Cr show a decrease change and Ni shows a zigzag change; Cu and Zn show a decreasing-increasing-decreasing-increasing trend. Corg, N, TC and TFe_2_O_3_ in the profile soil have a very significant impact on the vertical distribution characteristics of heavy metals. There are certain differences in the pollution degree of heavy metals in the surface soil of different profiles. Except for profile HCB01, where Cd and Hg in the surface soil are at moderate pollution levels, the heavy metals in the surface soil of the other profiles are at non-pollution to mild pollution levels. Principal component analysis results show that As, Cr, Cu and Ni belong to natural sources, Cd and Hg belong to anthropogenic sources from agricultural activities-atmospheric deposition, and Pb and Zn have both sources.

## Introduction

Black soil is a unique treasure given by nature to human beings. It has the characteristics of loose texture, high fertility and strong fertilizer supply ability, and is called the “giant panda” in soil. It is the cornerstone of the grain warehouse in Northeast China and the land foundation of agriculture in Northeast China [1–3]. In recent years, with the acceleration of urbanization and the continuous emergence of incorrect land cultivation, heavy metal elements have been accumulated in black soil [3–5]. The accumulation of heavy metals in soil affects the physical and chemical properties of soil, leading to the imbalance of soil nutrient circulation, which directly affects the productivity of soil and leads to the decline of crop yield and quality [6–9]. In addition, heavy metal pollution has a wide range, long duration, easy accumulation and difficult decomposition, and can pose a threat to human health through direct contact or food chain transmission [10–13]. For example, the accumulation of mercury can lead to autoimmune diseases and lung and kidney failure. Long-term excessive intake of Cd leads to prostatic hyperplasia, fracture, renal dysfunction, lung cancer and lung adenocarcinoma and other adverse effects [14]. European countries such as Germany, the United Kingdom, Denmark, Spain, Italy, the Netherlands and Finland were reported to have about 400000 soil heavy metal contamination sites, Sweden, France, Hungary, Slovakia and Austria have about 200000 sites, Greece and Poland have reported 10000 contaminated land, Ireland and Portugal have about 10000 contaminated sites, and the United States has about 6100000 hm^2^ of brown soil contaminated by heavy metals [15]. The total over standard rate of soil in China was 16.1%, and the proportions of slight, mild, moderate and severe pollution sites were 11.2%, 2.3%, 1.5% and 1.1%, respectively, the over standard rate of point for Cd, Hg, As, Cu, Pb, Cr, Zn and Ni were 7.0%, 1.6%, 2.7%, 2.1%, 1.5%, 1.1%, 0.9% and 4.8% respectively [16]. It is of great significance to study the content of heavy metals in soil and assess their risks to avoid the harm of heavy metals to the ecological environment and human health.

In recent years, domestic and foreign scholars have carried out studies on heavy metal pollution and ecological risk assessment of black soil under different conditions such as natural conditions, industrial, mining and transportation development in different regions. The average concentration of Cu, Cr and Cd in the black soil area of Changchun, Jilin Province was 1.15 times, 1.22 times and 1.19 times of the background value, respectively, which mainly came from agricultural production and industrial transportation [17]. In the black soil area in the middle of Heilongjiang Province, 3% of the Cd sites exceeded the screening value of agricultural soil pollution risk, and the Cd pollution rate was as high as 41% [18]. The average contents of Cd and Pb in the surface soil of Belgorodezhou black soil region of Russia were 0.20μg/g and 13.1μg/g, and the concentrations of Cd and Pb in mobile forms were 0.08μg/g and 1.09μg/g [19, 20]. In general, the study of heavy metals in black soil mainly focuses on the surface soil, but there are few reports on the distribution characteristics of heavy metals in black soil profile.

Haicheng City is located in the south-central part of Liaoning Province and the central part of Anshan City. It is located on the left bank of the lower reaches of the Liaohe River and the northern end of the Liaodong Peninsula. Haicheng City is not only one of the 17 key counties in typical black soil areas that have designated black soil cultivated land protection zones, it is also an important mineral development zone in the country. With the progress of the times and the development and changes of society, Haicheng City is dominated by the petrochemical industry and mining industry. Resource consumption problems and geological environmental problems caused by energy-intensive industries have become increasingly prominent. At the same time, with high-intensity agricultural farming, the accumulation of heavy metals in cultivated land on both sides of the river in Haicheng City has become increasingly intensified, especially the most significant enrichment of Cd and Hg. At present, the research on the vertical distribution of heavy metals and their ecological risks in soil profiles in this region is not comprehensive enough. In this paper, 8 heavy metals (As, Cd, Cr, Cu, Zn, Hg, Ni, Pb) in 93 soil samples from 5 sections in Haicheng City were selected as objects, and the vertical distribution characteristics of heavy metals in black soil were studied by geological accumulation index and ecological risk index. To explore the relationship between heavy metals and soil nutrient elements in the black soil of Haicheng City, as well as the pollution degree and potential ecological risks, in order to provide a basis for the safe utilization of soil resources and the prevention and restoration of soil pollution by local governments.

## Material and methods

### Study area

**Fig 1:**
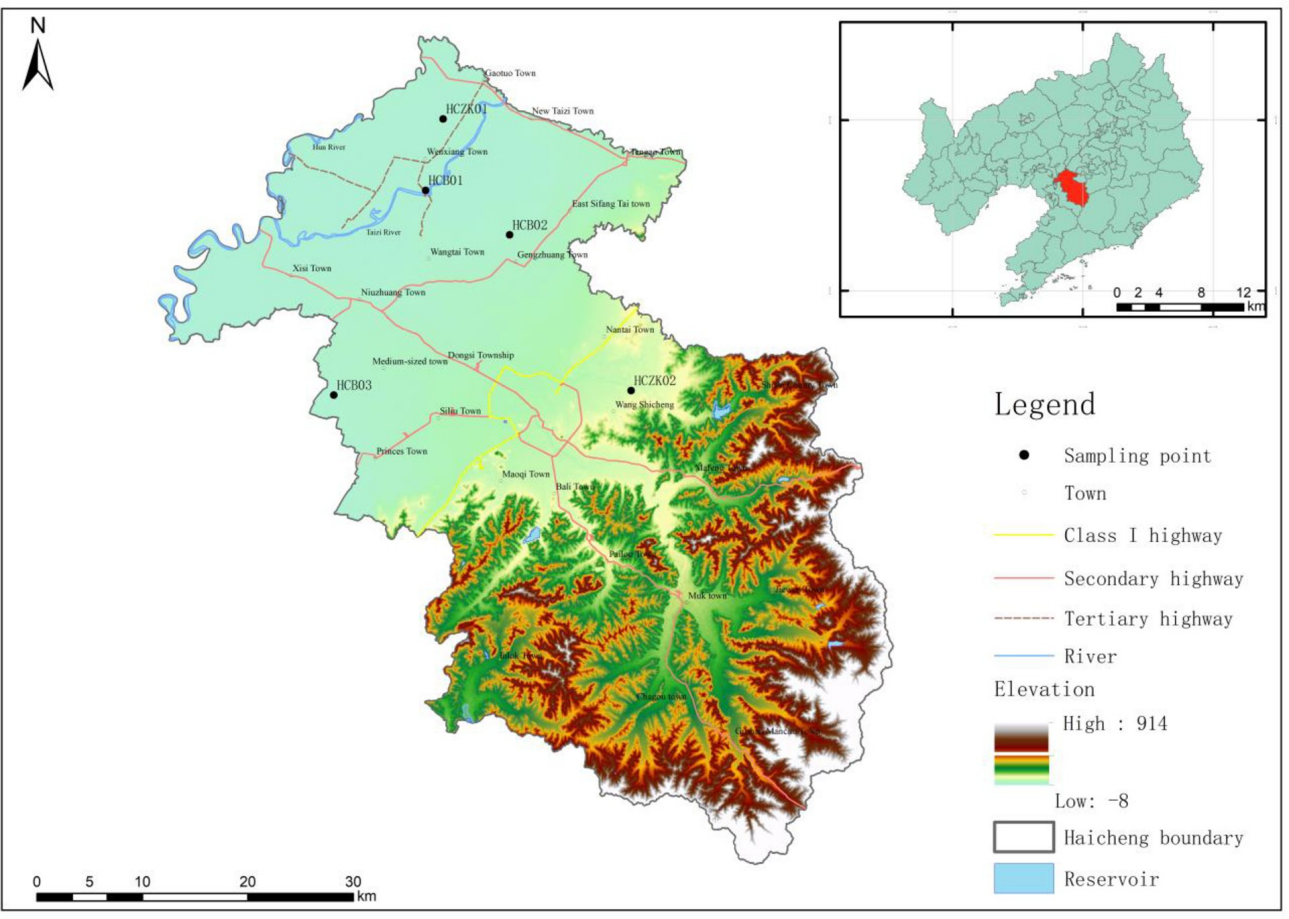
Locations of study area and sampling sites (Esri reserves the right to grant permission for any other use of the image).

Haicheng City located in the central south of Liaoning Province, the middle of Anshan city, is located in the lower left bank of the Liaohe River, the north end of the Liaodong Peninsula. It is located at 40°29’-41°11′N and 122°18′-123°08′E (Fig 1). The city has a total area of 2732km^2^ and an altitude of 60-500m .It is in the warm temperate monsoon climate zone, with an average annual temperature of 10.4°C and an average annual rainfall of 721.3mm. Haicheng city has Taizi River, Hunhe River, Daliao River in the north and south; The Haicheng River, the Wudao River, the Sandong River, the Yangliu River and the Eight Li River run across the east and west Agricultural production activities in the study area are developed, the main crops are five meters, corn, soybeans, wheat, and some vegetables and fruits are interspersed. Farm manure and conventional fertilizers are usually used.

### Sample collection and measurement

Five soil profiles (HCB01, HCB02, HCB03, HCZK01, HCZK02) were set up at five sampling points. HCB01, HCB02 and HCB03 were sampled using a backpack drill with a sampling depth of 500mm, while HCZK01 and HCZK02 were sampled using a car drill with a sampling depth of 5000mm for HCZK01 and 4100mm for HCZK02. On-site sample collection has been approved by local managers. A total of 93 soil samples were collected from the five soil profiles, samples were collected at different depth intervals. Locate sampling points with portable GPS. Visible impurities were removed from all collected samples and then air-dried at room temperature. Sample collection completed in 2023. The sampling interval and the description of the sampling points is shown in Table 1.

**Table 1:** Sampling points of different soil column profiles in the study area.

Sampling point	Coordinate		Sampling depth(mm)	sampling interval(mm)	Number of samples
	E	N			
HCB01	122.5985	41.042794	500	0–30, 30–100, 100–150, 150–200, 200–250, 250–400, 400–500	7
HCB02	122.6949	41.007731	500		7
HCB03	122.5044	40.865319	500		7
HCZK01	122.615147	41.104246	5000	0–15, 15–59, 59–110, 110–190, 190–265, 265–425, 425–530, 530–852, 852–940, 940–1200, 1200–1405, 1405–1515, 1515–1560, 1560–1600, 1600–1682, 1682–1740, 1740–1815, 1815–2180, 2180–2450, 2450–2650, 2650–2850, 2850–3000, 3000–3250, 3250–3500, 3500–3615, 3615–3700, 3700–3752, 3752–3800, 3800–3925, 3925–3973, 3973–4770, 4770–5000	32
HCZK02	122.837923	40.878759	5000	0–50, 50–120, 120–192, 192–265, 265–362, 362–460, 460–550, 550–640, 640–692, 692–745, 745–808, 808–866, 866–956, 956–1046, 1046–1100, 1100–1181, 1181–1262, 1262–1381, 1381–1500, 1500–1610, 1610–1714, 1714–1834, 1834–1940, 1940–2037, 2037–2175, 2175–2245, 2245–2400, 2400–2550, 2550–2660, 2660–2862, 2862–2970, 2970–3100, 3100–3260, 3260–3420, 3420–3600, 3600–3700, 3700–3800, 3800–3900, 3900–4000, 4000–4100	40

Based on the “Specifications for Geochemical Evaluation of Land Quality” (DZ/T0295-2016), C_org_ is measured by volumetric method (analysis methods for regional geochemical sample-part 27: determination of organic carbon contents by potassium dichromate volumetric method (DZ/T0279.27–2016)). N is measured by combustion infrared method (analysis methods for regional geochemical sample-part 29: determination of nitrogen contents by Kjeldahl distillation-volumetric method (DZ/T0279.29–2016)). As and Hg are measured by atomic fluorescence method (methods for chemical analysis of silicate rocks-part 33: determination of arsenic, stibium, bismuth and mercury elements content-hydride generation atomic fluorescence spectrometry (GB/T 14506.33–2019)). Cu, Pb, Zn, Ni, Cr, P, Cd, K_2_O, S and Sc are measured by X-ray Fluorescence (analysis methods for regional geochemical sample-part 1: determination of 24 components including aluminum oxide etc. by pressed powerpellets-X-ray fluorescence spectrometry (DZ/T0279.1–2016)), inductively coupled plasma mass spectrometry (analysis methods for regional geochemical sample-part 3: determination of 15 elements including barium, beryllium, bismuth etc. by inductively coupled plasma mass spectrometry (DZ/T0279.3–2016)) and inductively coupled plasma optical emission spectrometer (analysis methods for regional geochemical sample-part 2: determination of 27 components including calcium oxide etc. by inductively coupled plasma atomic emission spectrometry (DZ/T0279.2–2016)). The quality of analysis and testing is controlled by means of inserting national first-level soil standard materials, repeatability inspection, abnormal point inspection and blank test.

### Pollution degree and enrichment factor method

Geo-accumulation index (I_geo_) was used to evaluate the degree of soil heavy metal pollution [21]. The enrichment factor (EF) is a useful index to distinguish between natural and anthropogenic sources of heavy metals. EF can be calculated based on the following functions [22]. The calculation formula of I_geo_ is as follows:

$${\text{I}}_{\text{geo}}=\log_{2}\left(\frac{C_{i}}{K\times B_{i}}\right)$$ (1)

where I_geo_ is the soil accumulation index of heavy metal *i*; C*i* is the measured value of soil heavy metal *i*; B*i* is the reference value, and the Soil background value in Liaoning province is selected [23] (Table 2); *k* is the correction coefficient, generally 1.5. The pollution degree of *I*_*geo*_ can be divided into 7 grades: I_geo_ < 0, 0 ≤ I_geo_ < 1, 1 ≤ I_geo_ < 2, 2 ≤ I_geo_ < 3, 3 ≤ I_geo_ < 4, 4 ≤ I_geo_ < 5 and I_geo_ ≤ 5 correspond to unpolluted, mild polluted, moderate polluted, moderate-heavy polluted, heavy polluted, heavy-extreme polluted and extremely heavy polluted, respectively.

$$\text{EF}={\left[{\text{M}}_{i}/{\text{M}}_{Sc}\right]}_{\text{S}}/{\left[{\text{M}}_{i}/{\text{M}}_{Sc}\right]}_{\text{UCC}}$$ (2)

where [M_*i*_/M_*Sc*_]_S_ is the concentration ratio of the heavy metal *i* to Sc in samples, while [M_*i*_/ M_*Sc*_] _UCC_ is the ratio of upper continental crust(UCC). Sc is a trace element, and has no significant anthropogenic sources, so Sc is chosen as the reference element [22]. Generally, according EF value the soils can be classified as deficiencyto minimal enrichment (<1), mild enrichment (1–2), moderate enrichment (2–5), significant enrichment (5–20), very high enrichment (20–40), or extremely high enrichment (≥40).

**Table 2:** Descriptive statistical results of soil composition.

Soil Profile	Item	As	Cd	Cr	Cu	Hg	Ni	Pb	Zn	Corg	N	P	S	Sc	TC	TFe_2_O_3_
		μg/g	μg/g	μg/g	μg/g	μg/g	μg/g	μg/g	μg/g	%	μg/g	μg/g	μg/g	μg/g	%	%
HCB01	Topsoil content	11.3	0.38	80.7	34.1	0.20	32.1	45.6	158	1.09	710	857	204	12.1	1.24	10.4
	Max	11.3	0.38	93.8	34.1	0.20	38.7	45.6	158	1.38	993	881	243	15.8	1.51	10.4
	Min	4.78	0.086	56.8	12.6	0.018	22.1	20.6	60.4	0.44	424	544	115	8.83	0.48	4.66
	Mean(SD)	7.95(1.88)	0.16(0.11)	78.5(10.7)	25(6.70)	0.074(0.072)	31.7(4.94)	29.6(8.79)	94.5(34.1)	0.79(0.31)	705(178)	719(128)	188(44.1)	12.6(2.06)	0.86(0.35)	6.50(2.01)
HCB02	Topsoil content	12.3	0.15	104	32.7	0.041	45.6	33.6	91.0	1.61	1579	573	244	18.2	1.68	7.01
	Max	14.5	0.15	114	33.2	0.041	50.8	33.6	91.0	1.61	1579	573	244	18.2	1.68	7.96
	Min	8.48	0.052	73.4	21.3	0.011	30.2	23.0	56.9	0.15	214	262	71.1	12.5	0.18	4.39
	Mean(SD)	11.4(1.84)	0.084(0.031)	88.3(13.8)	25(5.04)	0.02(0.01)	37.1(7.34)	26.3(3.64)	69.9(13.3)	0.53(0.56)	564(494)	418(91.6)	123(61.5)	14.8(2.18)	0.71(0.51)	5.64(1.22)
HCB03	Topsoil content	16.5	0.24	96.8	33.0	0.037	43.0	34.4	133	1.63	1668	756	325	17.6	1.77	6.83
	Max	20.8	0.24	106	38.5	0.037	47.0	34.4	133	2.66	1668	756	9735	20.6	2.70	8.41
	Min	6.00	0.065	70.6	15.2	0.012	25.0	23.4	53.4	0.21	203	314	103	12.7	0.24	3.60
	Mean(SD)	13.5(4.94)	0.12(0.055)	93.8(13)	30.1(9.02)	0.024(0.0079)	39.8(9.16)	29.9(3.8)	90.6(26.1)	1.04(0.80)	900(546)	592(161)	1582(3330)	17.7(3.28)	1.11(0.81)	6.29(1.75)
HCZK01	Topsoil content	9.10	0.14	87.6	36.1	0.031	35.6	28.5	91.1	1.02	1165	785	257	13.2	1.12	6.39
	Max	10.4	0.15	96.4	42.2	0.031	41.4	29.8	93.9	1.16	1165	838	330	14.5	1.17	7.44
	Min	0.71	0.018	14.2	0.60	0.008	5.24	0.50	10.8	0.06	41.3	113	57.8	0.60	0.10	0.84
	Mean(SD)	3(2.96)	0.047(0.036)	33.7(24.9)	10.6(11.8)	0.013(0.0072)	12.8(10.8)	15.5(5.81)	28.3(26.2)	0.23(0.28)	246(322)	285(219)	128(65.2)	4.76(3.98)	0.26(0.30)	2.14(1.97)
HCZK02	Topsoil content	7.74	0.18	75.4	20.6	0.083	27.8	29.4	56.8	1.32	1241	452	196	9.25	1.41	4.17
	Max	21.5	0.24	95.2	43.1	0.083	46.1	35.3	102	1.32	1241	5102	196	13.9	1.41	9.02
	Min	0.79	0.034	30.2	12.4	0.003	15.1	17.2	20.4	0.051	62	223	42.2	5.01	0.100	2.46
	Mean(SD)	7.20(5.13)	0.079(0.039)	69.4(19.4)	26.2(6.68)	0.016(0.014)	31.6(6.46)	23.9(4.33)	60.4(20.6)	0.17(0.20)	238(191)	1225(1638)	91.7(35.8)	11.0(2.11)	0.19(0.21)	5.42(1.69)
Background value	Liaoning province	8.8	0.108	57.9	19.8	0.037	25.6	21.1	63.5	/	/	/	/	/	/	/
	China	11	0.097	61	23	0.065	27	26	74	/	/	/	/	/	/	/

### Data analysis

Descriptive statistics were implemented in SPSS 26 and Microsoft Excel 2010. Using Origin 2019b to carry out drawing. Pearson correlation analysis reveals the relationship between heavy metals.

## Results and discussion

### Soil basic properties in the study area

The contents and physicochemical properties of soil heavy metals in the study area are shown in Table 2. The content ranges of Corg, N, P, S, Sc, TC, and TFe_2_O_3_ in the topsoil soil of the five profile sampling points are 1.02%-1.63%, 710–1668μg/g, 452–857μg/g, 196–325μg/g, 9.25–18.2μg/g, 1.12%-1.77% and 4.17%-10.4%, respectively. The content ranges of As, Cd, Cr, Cu, Hg, Ni, Pb and Zn in the surface soil of the five profile sampling points are 7.74–16.5μg/g, 0.14–0.38μg/g, 75.4–104μg/g, 20.6–36.1μg/g, 0.031–0.20μg/g, 27.8–45.6μg/g, 28.5–45.6μg/g and 56.8–158μg/g, respectively. In the five surface soils, Cd, Cr, Ni and Pb exceeded the background values of Liaoning and China soils. Except for Cu in the surface soil of HCZK02, which was lower than the background value of China soil, the others were higher than the background values of Liaoning and China soils. For As and Zn, except for the content in the surface soil of HCZK02, which was lower than the corresponding background values of Liaoning and China soils, and the As content in the surface soil of HCZK02, which was lower than the background value of China soils, the others were higher than the background values of Liaoning and China soils. For Hg, the content in the surface soil of profiles HCB01 and HCZK02 was higher than the two soil background values, the content in the surface soil of profiles HCB03 and HCZK01 was not higher than the two soil background values, and the content in the surface soil of HCB02 was higher than the Liaoning soil background value and lower than the China soil background value. This means that human activities such as irrigation, fertilization, and transportation have had a certain impact on soil heavy metals in the study area.

In profile HCB01, the content ranges of As, Cd, Cr, Cu, Hg, Ni, Pb, and Zn are 4.78–11.3, 0.086–0.38, 56.8–93.8, 12.6–34.1, 0.018–0.20, 22.1–38.7, 20.6–45.6μg/g, and 60.4–158μg/g, respectively. In profile HCB02, the content ranges of As, Cd, Cr, Cu, Hg, Ni, Pb, and Zn are 8.48–14.5, 0.052–0.15, 73.4–114, 21.3–33.2, 0.011–0.041, 30.2–50.8, 23–33.6μg/g, and 56.9–91.0μg/g, respectively. In profile HCB03, the content ranges of As, Cd, Cr, Cu, Hg, Ni, Pb, and Zn are 6.00–20.8, 0.065–0.24, 70.6–106, 15.2–38.5, 0.012–0.037, 25–47, 23.4–34.4μg/g, and 53.4–133μg/g, respectively. In profile HCZK01, the content ranges of As, Cd, Cr, Cu, Hg, Ni, Pb, and Zn are 0.71–10.4, 0.018–0.15, 14.2–96.4, 0.6–42.2, 0.008–0.031, 5.24–41.4, 0.5–29.8μg/g, and 10.8–93.9μg/g, respectively. In profile HCZK02, the content ranges of As, Cd, Cr, Cu, Hg, Ni, Pb, and Zn are 0.79–21.5, 0.034–0.24, 30.2–95.2, 12.4–43.1, 0.0030–0.083, 15.1–46.1, 17.2–35.3μg/g, and 20.4–102μg/g, respectively. It can be seen that the deeper the profile depth, the greater the range of changes in heavy metals.

### Vertical distribution of heavy metals in soil profile

**Fig 2:**
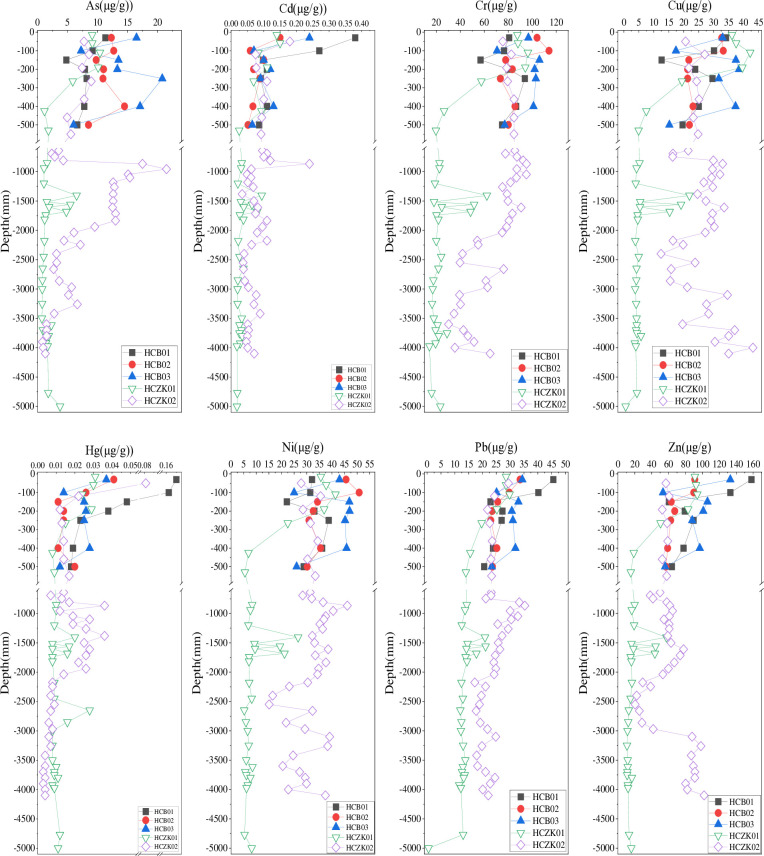
Vertical distribution characteristics of heavy metals in soil profiles at five sampling points.

**Fig 3:**
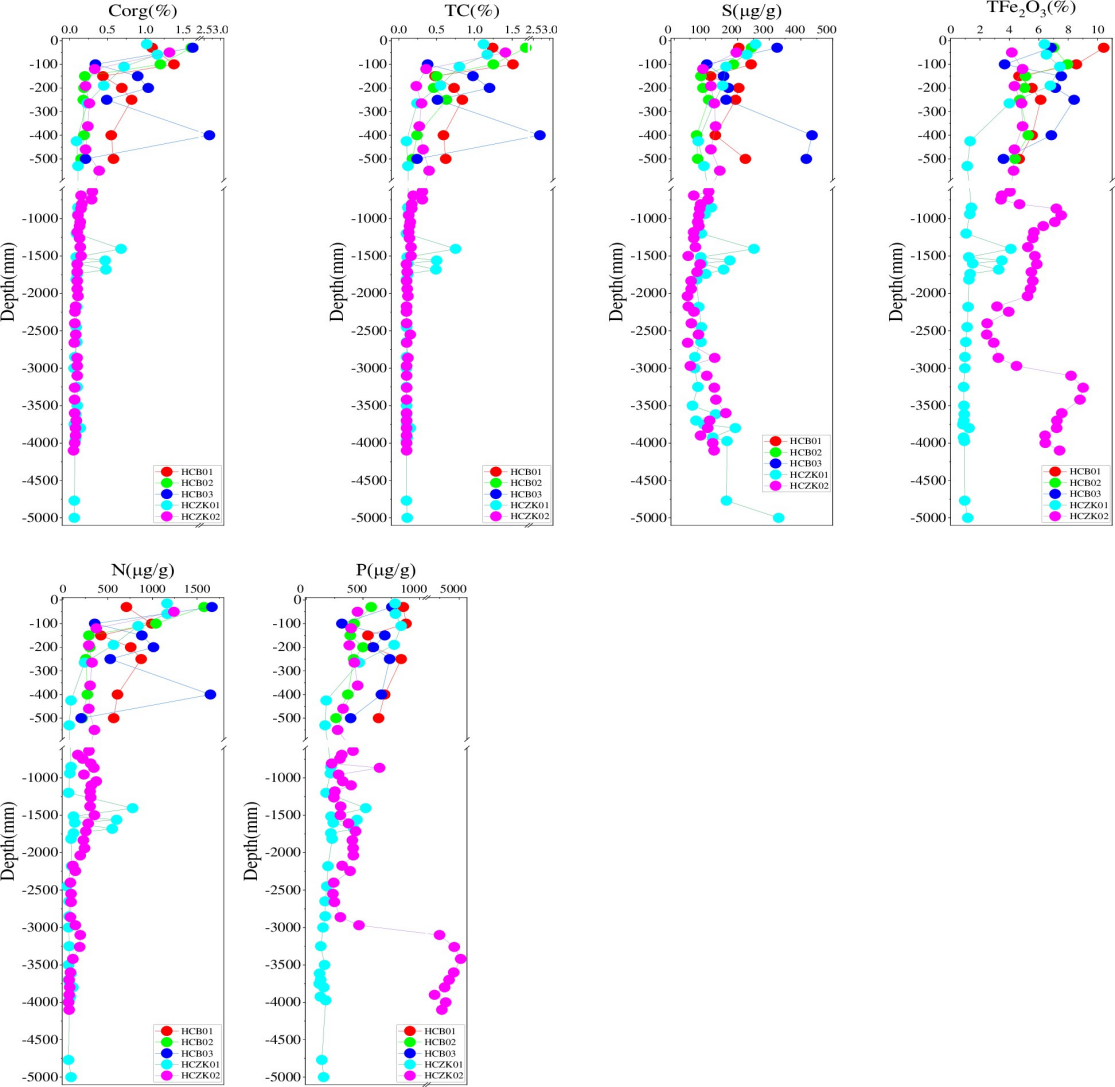
Vertical distribution characteristics of organic carbon (Corg), total carbon (TC), S, total iron (TFe_2_O_3_) and nutrient elements in soil profiles at five sampling points.

The distribution characteristics of soil heavy metal elements As, Cd, Cu, Hg, Ni, Pb and Zn in the five profiles are shown in Fig 2. In profiles HCB01, HCB01, HCB01 and HCZK01, the contents of As, Cd, Cu, Hg, Ni, Pb and Zn generally decreased with increasing depth, which may be caused by natural changes [24]. The high content of heavy metals in the surface soil further indicates that human activities will cause certain disturbance to the soil in the study area, and some heavy metals will gradually accumulate in the surface soil with industrial activities, fertilizer application, pesticide application and automobile exhaust emissions [3, 24, 25]. Interestingly, when the depth of profile HCZK01 was 1200–1600 mm, the contents of the eight heavy metal elements all showed a sawtooth change phenomenon of increase-decrease-increase-decrease. At the depth of 1200-1600mm, the parent material components Al_2_O_3_, CaO, MgO, Mn, TFe_2_O_3_ (Fig 3) and Ti also show zigzag changes, suggesting that the zigzag changes of heavy metals may be influenced by the parent material [13, 26]. Liao et al. [27] studied the typical soil profile of Muchuan County, Sichuan Province, and found similar distribution characteristics of heavy metals. However, Zhou et al. [28] study on Sc, V, Co, Ni, Mo and Ba in soil profiles of the Men River Basin in northeast Thailand showed that heavy metal content in most soil profiles generally increased with depth. The vertical distribution characteristics of heavy metals in profile HCZK02 were more complex. The contents of As, Hg and Pb showed a decrease-increase-decrease change with increasing depth; the contents of Cd and Cr generally decreased with increasing depth; the content of Ni showed a sawtooth change with increasing depth; the contents of Cu and Zn showed a decrease-increase-decrease-increase trend with increasing depth, and the contents of Cu and Zn in deep soil were significantly higher than those in upper soil. It is speculated that this change is mainly caused by the soil parent material [29–31]. Taking Cu and Zn as an example, the vertical distribution characteristics of Cu and Zn are very similar to the vertical distribution characteristics of TFe_2_O_3_ (Fig 3), and correlation analysis shows that Cu and Zn are significantly positively correlated with TFe_2_O_3_, and the correlation is 0.725 and 0.898, respectively. This is attributed to the adsorption of these heavy metals by iron and manganese oxides [26, 32], which have strong adsorption and enrichment capacity for heavy metals in soil due to the formation of surface complexes and the existence of specific adsorption potentials [33, 34]. However, others did not show positive concentration peaks in this layer, suggesting that the effect of iron manganese oxides on these heavy metals is limited. The underlying reasons for this need to be further explored. The vertical distribution of heavy metal concentration can reflect the relative migration of heavy metals in the soil profile [35]. According to the distribution characteristics of these heavy metals in the five profiles (Fig 2), it can be concluded that the mobility of heavy metals As, Cd, Hg, Pb and Zn in these soil profiles is higher than that of Cr, Cu and Ni. The five soil profiles were excavated in agricultural soils where fertilizer was frequently used. The high concentration of heavy metals in the surface soil of the profile was caused by the application of pesticides and fertilizers [27, 28]. Unlike shallow soil, deep soil has less interaction with external heavy metal sources, and heavy metals in deep soil are also largely related to the parent material of historical periods.

### Vertical distribution of Corg, TC, S, TFe_2_O_3_ and nutrient elements in soil profile

The vertical distribution of soil Corg, TC, S, TFe_2_O_3_, N and P in the five profiles were shown in Fig 3. Soil Corg content ranged from 0.051% to 2.66%, and soil TC content ranged from 0.10% to 2.70%. The vertical distribution characteristics of Corg and TC were consistent and decreased with the increase of soil depth. The content of S soil varied from 42.2 to 9735μg/g, with a large span, and the maximum content was 230 times of the minimum content. S content decreased first and then increased with the increase of soil depth, and this change was more significant in HCB03, HCZK01 and HCZK02.

The TFe_2_O_3_ content ranges from 0.84%-10.4%, with the most significant changes observed in profile HCZK02, and a significant increase in content below a depth of 2970mm. The N content ranges from 41.3–1668μg/g and decreases with increasing soil depth. The P content ranges from 113–51020μg/g and decreases with increasing soil depth in profiles HCB01, HCB02, HCB03, and HCZK01; however, there is a significant difference in profile HCZK02, with a significant increase in content below a depth of 2970mm. The P content at a depth of 2862-2970mm is 465μg/g, while at a depth of 2970-3100mm, the content increases to 3745μg/g.

In general, Corg, TC, S, TFe_2_O_3_ and nutrient elements decreased sharply from the surface layer to the subsurface layer, decreased significantly in the 40–200 cm soil layer, and tended to be stable in the soil layer below 300 cm (Fig 3). Unlike shallow soils, deep soils interact less with the atmosphere, hydrosphere, and biosphere, resulting in less exchange of matter and energy [36]. In deep soils, Corg,TC, S, TFe_2_O_3_ and nutrient elements are also largely associated with the parent material of historical periods. Since the parent material is closely related to the mineralogy, type and particle composition of the soil, it may be related to the stabilization of Corg, TC, and S and nutrient elements [37, 38]. Deep soils usually have low porosity, high bulk density [39], and high clay content [40], which hinder the migration of Corg, TC, S and nutrient elements. Helps to make deep soil with Corg, TC, S and nutrient elements more stable.

### Contamination assessment by I_geo_ values in topsoil

**Fig 4:**
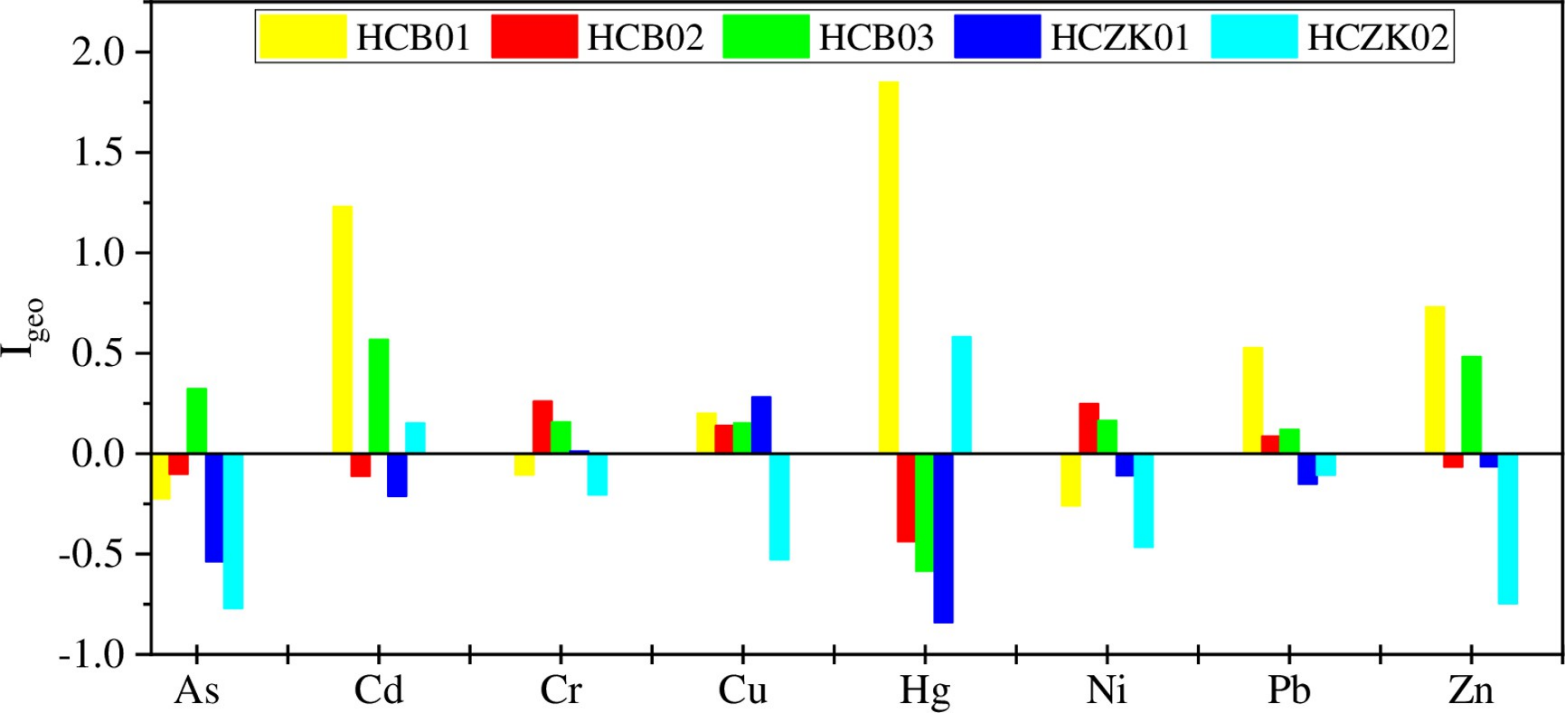
Geoaccumulation indexes of heavy metals in the topsoil of five soil profiles.

Fig 4 shows the I_geo_ values of heavy metals in the surface soil of the five profiles. It can be seen that there are certain differences in the pollution of heavy metals in the surface soil of different profiles. In profile HCB01, the I_geo_ values of heavy metals As, Cr and Ni are all less than 0, while the I_geo_ values of Cd, Cu, Hg, Pb and Zn are all above 0, especially the I_geo_ values of Cd and Hg are 1.23 and 1.85, respectively. This indicates that the heavy metals in profile HCB01 are not polluted by As, Cr and Ni, but are mildly polluted by Cu, Pb and Zn, and moderately polluted by Cd and Hg. The surface soil of profile HCB02 was not contaminated by heavy metals As, Cd, Hg and Zn (I_geo_ < 0), but heavy metals Cr, Cu, Ni and Pb were mildly contaminated (0 ≤ I_geo_ < 1). For the surface soil of profile HCB03, except for Hg, the other 7 heavy metals were in a mildly polluted state (0≤I_geo_<1), and Hg was in a non-polluted state (I_geo_<0). In profile HCZK01, only Cu is in a mildly polluted state, and the other seven heavy metals are at a non-polluting level. For the surface soil of profile HCZK01, Cd and Hg were at a mildly polluted level, and the other 6 heavy metals were all in a non-polluted state. In general, since the five profile points are all agricultural soil, and the main crops are corn and rice, agricultural activities will inevitably bring different degrees of soil heavy metal pollution [2–4, 17, 41]. The study found that the use of fertilizers and manure increased the content of heavy metals (Cd, Pb and Zn) by about 3% per year [42]. Many studies have shown that the application of high-content Cd phosphate fertilizer is an important factor causing Cd enrichment in soil [43–45]. In addition, atmospheric deposition may also cause an important factor of heavy metal pollution in surface soil. As China’s old industrial base, Northeast China’s industrial production has grown rapidly in recent years [46–49]. Heavy metals produced by advantageous industries such as heavy industry, energy, agricultural and sideline food processing, textile industry, pharmaceutical manufacturing, non-metallic manufacturing, and transportation equipment manufacturing have been transported and deposited into the soil through the atmosphere, resulting in soil heavy metal pollution [49].

### Enrichment of heavy metals

**Fig 5:**
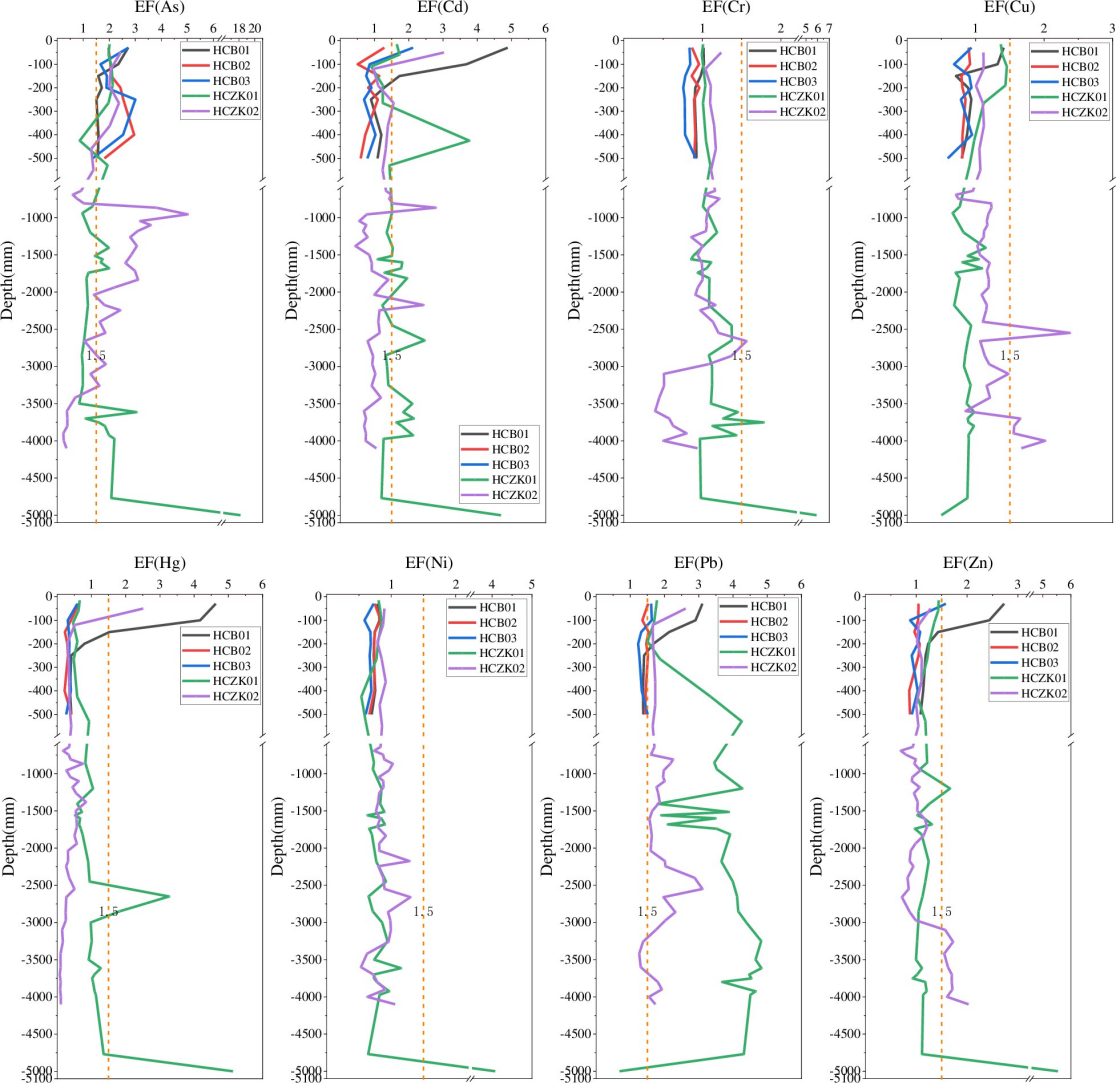
Heavy metal EF values in five soil profiles.

The EF values of heavy metals in the soil of the five profiles are shown in Fig 5. Most samples calculated EF factor values of less than 2, indicating minimal enrichment of these heavy metals in the soil [50]. Sources of heavy metals in soil profiles include crustal materials (such as parent rocks) and non-crustal materials (such as anthropogenic sources). Due to rock formation, EF = 1.5 is considered an important threshold for assessing whether human activities affect soil heavy metal concentrations [51]. The EF values of Cr, Cu and Ni in five profiles were all lower than 1.5, while the EF values of other heavy metals in one or several profiles were higher than 1.5. For example EF values of Cd and Hg above 3.5 in soil profile HCB01 appeared precisely in the upper surface soil, consistent with the corresponding I_ego_ results (Fig 5), which were attributed to agricultural activities or atmospheric deposition. Several extremely abnormal EF values appeared for Cr, Cu, Hg, Ni and Zn at different depths in the profile soil, which were exactly at the depths where the heavy metal concentrations appeared abnormal (Fig 2); while the EF values of As, Cd and Pb at different depths in the profile soil were mostly greater than 1.5, especially Pb. The heavy metal EF values greater than 1.5 appeared in the soil depth of 0-300mm, which was mainly due to the heavy metal pollutants introduced into the surface soil by human activities, which were carried downward by irregular leaching water (especially large amounts of rainwater, irrigation water, etc.) [52]. The heavy metal EF greater than 1.5 below the depth of 300mm can be attributed to the absorption and enrichment of iron and manganese oxides, especially the EF value of Pb in the profiles HCZK01 and HCZK02, which was similar to the corresponding TFe_2_O_3_ distribution (Fig 3).

### Correlation between heavy metal concentration with soil Corg, TC, S, TFe_2_O_3_ and nutrients

**Fig 6:**
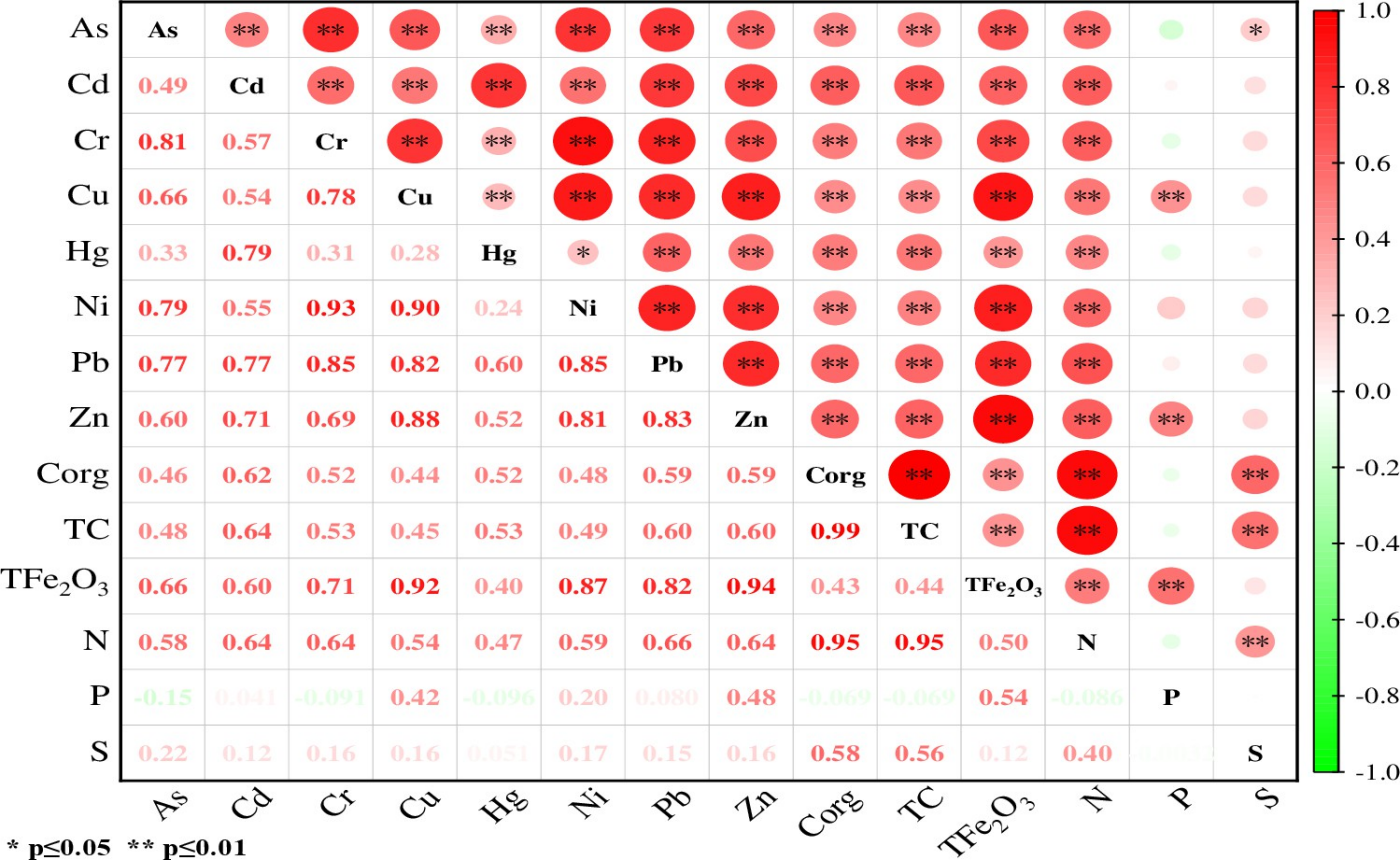
Pearson correlation between heavy metals in soil profiles with soil Corg, TC, S, TFe_2_O_3_ and nutrients at five sampling points.

Pearson correlation analysis was used to determine the correlation between profile soil heavy metals concentration with soil physicochemical components (Corg, TC, S, TFe_2_O_3_ and nutrients). Pearson correlation analysis can be used to determine the correlation between heavy metals and provide information about their source and transmission. If there is a significant positive correlation between heavy metals, it indicates that they come from the same source and have similar pathways [53, 54]. The correlation analysis results were shown in Fig 6. Except Hg-Ni showed significant correlation (p≤0.05, r = 0.24), other heavy metals showed extremely significant positive correlation (p≤0.01), indicating that the 8 heavy metals originated from similar natural processes and have similar geochemical behavior [55]. However, the correlation coefficients between Cd with As, Cr, Cu and Ni are all less than 0.60, and the correlation coefficients between Hg and other heavy metals except Pb are also less than 0.60, while the correlation coefficient of Cd-Hg is 0.79, indicating that Cd and Hg have other input sources in addition to natural processes.

In soil physicochemical components, 8 heavy metals showed significant positive correlation with Corg, N, TC and TFe_2_O_3_ (p≤0.01), Cu-P and Zn-P also showed significant positive correlation (p≤0.01), and As-S showed significant positive correlation (p≤0.05, r = 0.22). It is reported that the migration of heavy metals in soil is controlled by adsorption and desorption processes, which are affected by soil properties such as soil Corg content, TC content, N content and TFe_2_O_3_ content [56–58]. Black soil is rich in organic matter, Corg and N are the main components of soil organic matter, and soil organic matter contains a large number of organic ligands, which can absorb heavy metal ions through complexation or chelation [57], thereby regulating the migration capacity and toxicity of heavy metals. Nutrient elements such as carbon and nitrogen in soil can change the form, availability and migration ability of heavy metals by forming complexation or chelation with heavy metals [58]. Higher organic carbon content and total phosphorus content can increase the adsorption sites, chelating agents and phosphate precipitation in soil, and increase the oxidizable state of heavy metals [59]. The adsorption capacity of iron and manganese oxides to some toxic heavy metals has been confirmed by a large number of studies [60, 61]. Although the mass of iron and manganese oxides only accounts for a small part of the solid phase of soil, due to its high adsorption capacity, iron and manganese oxides often control the migration, adsorption, precipitation and other processes of heavy metal pollutants in soil, affecting the bioavailability of heavy metals [62].

### Principal component analysis for heavy metals

Principal component analysis (PCA) is an important multivariate analysis method used to identify the sources of heavy metals in soil [63]. The prerequisite for applying PCA is to pass KMO and Bartlett sphericity test: KMO value should be greater than 0.5, and Bartlett sphericity test should be significant (P<0.001) [50, 64]. The KMO value in this study was 0.843, and the Bartlett sphericity test was significant (P = 0.000), indicating that PCA was suitable for the analysis of these soil samples. The results of PCA are listed in Table 3.

**Table 3:** Principal component analysis (PCA) of heavy metals.

Element	Component	
	PC1	PC2
As	0.82	0.21
Cd	0.41	0.85
Cr	0.91	0.21
Cu	0.90	0.22
Hg	0.09	0.96
Ni	0.97	0.17
Pb	0.79	0.55
Zn	0.74	0.50
Variance (%)	57.57	29.73
Cumulative (%)	57.57	87.30
KMO	0.843	
P	0.000	

Factors with eigenvalues greater than 1 were selected by Kaiser, and two principal components (PC1 and PC2) were extracted. The cumulative contribution rate of the factors was 87.30%, and most of the information of 8 heavy metals could be analyzed. It is speculated that there are two main sources of heavy metals in the soil of the study area. The contribution rate of PC1 is 57.57%, and it is composed of As, Cr, Cu, Ni, Pb and Zn, with load coefficients ≥0.74, belonging to the strong load [65]. Correlation analysis showed that these six heavy metals had significant homology and similar geochemical behavior (Fig 3). Relevant studies show that As, Cr, Cu, Ni, Pb and Zn are mainly controlled by geological background and parent material [66]. It is speculated that the sources of As, Cr, Cu, Ni, Pb and Zn in PC1 are mainly natural sources.

The contribution rate of PC2 is 29.73%. Cd and Hg have extremely significant loads, which are 0.85 and 0.96 respectively. In addition, Pb and Zn also have obvious loads, which are 0.55 and 0.50 respectively. Correlation analysis shows that Cd and Hg have extremely significant loads. correlation. Relevant studies have shown that Cd in soil treated with pesticides is more than 200 times that in soil without pesticides [67]. The application of agricultural chemical fertilizers leads to the accumulation of Cd, Hg, Pb and Zn to a certain extent. Cd, Pb and Zn mostly exist in chemical fertilizers such as phosphate fertilizers. The application of agricultural chemical fertilizers will lead to the accumulation of soil Cd, Pb and Zn [68]. Organic matter is rich in functional groups with high affinity for Hg. Excessive application of organic fertilizers will lead to high levels of Cd and Hg in local areas [69]. Industrial production activities such as non-ferrous metal smelting [70] and plastic stabilizer production also produce Cd and Hg, which accumulate into the soil through atmospheric deposition along with dust [49]. It is speculated that the sources of Cd, Hg, Pb and Zn in PC2 in the study area are mainly agricultural sources and atmospheric deposition.

## Conclusions

The content, distribution characteristics and pollution level of heavy metals in 5 typical black soil profiles in Haicheng City, Liaoning Province were analyzed by geological accumulation index method and enrichment factor method. The contents of Cd, Cr, Ni and Pb in topsoil exceeded the background values of Liaoning and China. The contents of As, Cd, Cu, Hg, Ni, Pb and Zn in profiles HCB01, HCB01 and HCZK01 generally decrease with increasing depth. The profile distribution characteristics of As, Cd, Cu, Hg, Ni, Pb and Zn in HCZK02 are significantly different. There are some differences in the heavy metal pollution degree of surface soil in different profiles. EF values for Cr, Cu and Ni were below 1.5 in all five profiles, while EF values for other heavy metals were above 1.5 in one or more profiles. The distribution characteristics of heavy metals in the soil were positively correlated with Corg, N, TC, and TFe_2_O_3_, P has an extremely significant positive correlation with Cu and Zn, and As with S have a significant positive correlation. As, Cr, Cu and Ni are mainly controlled by geological background and parent material and belong to natural sources. Cadmium and mercury are anthropogenic sources, mainly agricultural sources and atmospheric deposits. Based on the research results, it is found that if the soil heavy metal pollution research is carried out, it is recommended to collect the upper layer of soil (0-300cm). In addition, it is recommended to conduct in-depth research on the accumulation characteristics of heavy metals in soil, rationally assess their ecological hazards, and ensure the safe use of land.
